# Endourologic Management of Upper Tract Transitional Cell Carcinoma following Cystectomy and Urinary Diversion

**DOI:** 10.1155/2009/976401

**Published:** 2008-12-28

**Authors:** Jeffrey John Tomaszewski, Marc Christopher Smaldone, Michael Cecil Ost

**Affiliations:** Department of Urology, University of Pittsburgh School of Medicine, Pittsburgh, PA 15213, USA

## Abstract

Traditionally, nephroureterectomy is the gold standard therapy for upper tract recurrence of transitional cell carcinoma (TCC) following cystectomy and urinary diversion. With advances in endoscopic equipment and improvements in technique, conservative endourologic management via a retrograde or antegrade approach is technically feasible with acceptable outcomes in patients with bilateral disease, solitary renal units, chronic renal insufficiency, or significant medical comorbidities. Contemporary studies have expanded the utility of these techniques to include low-grade, low-volume disease in patients with a normal contralateral kidney. The aim of this report is to review the current outcomes of conservative management for upper tract disease and discuss its application and relevance in patients following cystectomy with lower urinary tract reconstruction.

## 1. Introduction

 Upper tract transitional cell carcinoma (UTTCC) represents 5% of all urothelial cancers [1]. Due to the proposed field defect associated with these lesions, removal of the entire urothelium on the ipsilateral side offers the best chance of surgical cure. For this reason, the traditional and gold standard treatment for UTTCC has been radical nephroureterectomy [2]. However, minimally invasive endoscopic techniques have been developed to treat patients with bilateral upper tract disease, poor candidates for radical surgery, and those with solitary renal units. In more recent times, even healthy patients with low-grade, noninvasive tumors have been managed endoscopically, with the understanding that some may require radical nephroureterectomy if UTTCC should recur or progress.

The incidence of upper tract recurrence following radical cystectomy is low (3–5%), but there is an increased incidence of upper tract recurrence in patients undergoing cystectomy with a prior history of superficial bladder disease [3]. Treatment of upper tract recurrence following lower urinary tract reconstruction is challenging, but with recent technological advances, both ureteroscopic and percutaneous techniques have been utilized for surveillance and management in these complex patients. We review the literature in order to summarize and define the advantages and disadvantages of ureteroscopic and percutaneous management of upper tract TCC following urinary diversion.

## 2. Discussion

Upper tract transitional cell carcinoma (UTTCC) is relatively uncommon, accounting for approximately 5% of all urothelial tumors and 10% of all renal tumors or approximately 3000 cases per year in the United States [4, 5]. The incidence of upper tract recurrence following radical cystectomy for urothelial cancer ranges from 2% to 6% [3, 6–11], with the majority of recurrence in the first 3 years [3]. Additionally, Tran et al. [3] demonstrated that the risk of upper tract recurrence does not change with time, emphasizing the critical importance of continued surveillance for UTTCC following cystectomy. Patients with associated carcinoma in situ of the bladder or prostatic urethra, recurrent high risk superficial cancer of the bladder, and tumor multifocality are at higher risk of ureteral involvement at the time of cystectomy [3, 12–17]. In the subgroup with ureteral involvement at the time of cystectomy, tumor recurrence in the upper tract was noted in 16–17% [3, 16], with the authors concluding that these patients require vigorous follow up with urine cytology and upper tract surveillance imaging.

Treatment of upper tract recurrence following cystectomy remains a clinical dilemma. Due to improvements in fiberoptic technology and refinement of endoscopic techniques, conservative management of UTTCC has evolved into a viable treatment alternative with similar efficacy to that of radical therapy in select patients with noninvasive and low-grade disease. Indications for ureteroscopic and/or percutaneous endoscopic management include patients with a solitary kidney, bilateral disease, renal insufficiency, or patients who would require dialysis after nephroureterectomy [18–20]. However, in recent series, minimally invasive procedures have increasingly been utilized in patients with a normal contralateral kidney [21–23]. These studies have concluded that patients who have solitary, small (<1.5 cm), low-grade, and completely resectable tumors are candidates for endoscopic management if they are willing to accept lifelong surveillance for recurrence [21, 24].

Surveillance should be lifelong and tailored to the patient's tumor grade and stage. Our institution's surveillance protocol includes urine cytology every three months, and upper tract imaging (computed tomography urogram, intravenous pyelogram, or retrograde pyelogram) every six months for the first two years, then yearly thereafter [20, 25, 26]. Contemporary surveillance protocols for upper tract disease include surveillance ureteroscopy at frequent defined intervals [26, 27]. In patient's with lower urinary tract reconstruction, this may not be feasible and needs to be tailored to each individual patient. Management of recurrent upper tract TCC is comparable to that of primary upper tract TCC and must be adapted to the tumor characteristics and patient; nephroureterectomy is usually recommended for recurrences that have evidence of grade and stage progression.

A drawback of endoscopy in the management of pelvicaliceal lesions is its low sensitivity for the detection of invasive lesions and thus its low reliability in staging [25, 26, 28]. The correlation between grade and stage of upper tract tumors has previously been demonstrated, thus many rely primarily on the grade of the endoscopic biopsy specimen for pathologic assessment [26, 29]. Abnormal upper tract urinary cytology results have been shown to predict tumor recurrence and correlate with pathologic tumor grade and stage [26, 28, 30].

Surgical resection of a UTTCC following cystectomy and continent or incontinent urinary diversion presents a unique challenge. Although such a recurrence portends an overall poor prognosis, a maximal effort must be made to resect localized disease. Endoscopic management of upper tract abnormalities in patients following urinary diversion is complicated by difficult retrograde access to the upper collecting system [31]. Although technically challenging, endoscopic retrograde, percutaneous antegrade or combined antegrade, and retrograde approaches have been described [31, 32] and can be utilized in the evaluation and treatment of upper tract urothelial cancer recurrence.

## 3. Retrograde Ureteroscopic Access

The ureteroscopic approach is typically the least invasive surgical treatment option for UTTCC. It is also the most thorough procedure for surveying the entire collecting system for posttreatment surveillance. Advantages include limited morbidity in the setting of an outpatient procedure and the potential oncologic benefit of a closed system [31]. The most challenging aspect of ureteroscopic management following both continent and incontinent lower urinary tract reconstruction is obtaining retrograde access to the ureter.

In cases following continent diversion, the neobladder can be accessed via the urethra using rigid or flexible ureteroscopy [33]. In cases of incontinent urinary diversion, a flexible cystoscope can be passed through the stoma into the reservoir. Mucous and debris is often encountered on initial inspection and must be copiously irrigated to improve visualization [31]. A cystogram or loopogram under fluoroscopic guidance can be performed to help delineating the afferent limb and the ureteral anastomosis. Administration of methylene blue or indigo carmine may aid in the identification of the ureteral anastamoses. Upon identification, the ureteral orifices can be cannulated with guidewires or open-ended ureteral access catheters. The use of contrast to clearly delineate anatomic landmarks can also facilitate a combined antegrade/retrograde approach in select mid-ureteral tumors that may require dual access for complete resection. In select cases ureteral access sheaths can help to facilitate repeated passes of the ureteroscope and tumor basketing. In addition, the use of an access sheath decreases irrigation pressure [34] and may theoretically reduce the possibility of pyelolymphatic backflow and tumor dissemination. Baskets or biopsy forceps can be used for tumor debulking and biopsy, and electrocautery or laser fibers can be used to ablate tumor and control hemorrhage [33]. Disadvantages include potential staging errors, inability to treat large lesions in a single setting, and difficulty in accessing lower pole lesions [35].

Although minimally invasive treatment methods were originally developed for patients that could not undergo open surgery, the ureteroscopic approach to UTTCC has been shown to be an efficacious first-line treatment to address UTTCC of low stage and grade [35]. Ureteroscopy provides adequate access for biopsy under direct vision, and mechanical, ablative, or laser removal of papillary lesions anywhere along the upper tract urothelium. Chen and Bagley [36] treated 23 patients with UTTCC; 8 remained disease-free, and 15 had recurrences treated at a mean follow up of 35 months, with 100% disease-specific survival. Keeley et al. [26] and Martínez-Piñeiro et al. [19] reported tumor-free rates of 76% and 71%, respectively, among patients with low-grade UTTCC. Due to its efficacy, safety, and minimal morbidity, the ureteroscopic approach is a very attractive treatment alternative for low-grade urothelial carcinoma [33].

Nelson et al. [31] reported their experience with retrograde ureteroscopy for the management of 13 renal units in 8 patients following continent neobladder diversion. Indications for evaluation included upper tract filling defect, positive cytology, or renal calculi. The ureter and renal pelvis were successfully accessed and visualized in 76%, and they were unable to access the ureteral orifices in three remaining patients. While demonstrating that retrograde access is technically feasible in this patient population, attempting to access the collecting system retrograde in reconstructed patients may have severe consequences. Care must be taken to avoid damaging the continence mechanism, perforating the reservoir, or disrupting the ureteral-enteric anastomoses. In our practice, primary ureteroscopic therapy is considered for upper tract evaluation in patients with lower urinary tract reconstruction for small filling defects on upper tract imaging or positive cytology, with the intention of treating small lesions during the initial setting. All patients are counseled that access or treatment failure is a distinct possibility, and that further antegrade or more definitive open or laparoscopic surgical procedures may be warranted. Complications specific to ureteroscopic tumor treatment include extraluminal spillage or propagation of neoplasm, ureteral perforation, and ureteral stricture formation [22]. The reported stricture rate following ureteroscopic management of upper tract TCC has ranged from 5% to 14% [19, 26, 37]. When a ureteral stricture forms following endoscopic management of upper tract TCC, it is imperative to perform a biopsy of the region to rule out malignant disease [36].

## 4. Percutaneous Access

The evolution of lower urinary tract reconstruction has resulted in a growing number of patients in need of complex upper tract management. Although technically feasible, evaluation and treatment of upper tract abnormalities are complicated by difficult retrograde access to the upper collecting system due to unusual anatomy and lack of anatomic landmarks [31]. The difficulty of accessing both refluxing and nonrefluxing ureterointestinal anastamoses restricts the use of the size and type of endoscopic equipment necessary for complete resection of UTTCC which is challenging from a retrograde approach under ideal circumstances. Although more invasive, a percutaneous approach avoids these difficulties through direct access and offers a high success rate with minimal morbidity [32].

The method of obtaining percutaneous access is similar to what has been described for percutaneous nephrolithotomy. Under fluoroscopic guidance, a direct puncture of the involved calyx or an upper pole or central calyx puncture for renal pelvis, lower pole, or ureteral tumors is recommended [38]. Following tract dilation, a 30Fr access sheath is placed, and rigid or flexible nephroscopy may be performed. Once the offending lesion is visualized, frozen section pathology examination is recommended to rule out a high-grade lesion. The ideal resection modality depends on tumor size and type, but monopolar and bipolar cautery, laser, rollerball electrode, and electrovaporization techniques have been described. The entire tumor should be ablated and the base fulgurated or resected. Flexible nephroscopy should be carried out to ensure that all areas of the kidney are clear of tumor. A nephrostomy tube should be left in place for external drainage to preserve access and to facilitate adjuvant chemotherapy. In select cases, a second look nephroscopy on postoperative day 1-2 to ensure complete resection is recommended. In the case of continent cutaneous diversion, percutaneous access into the pouch under direct vision with a 10 mm trocar has also been described [39]. Historically utilized in cases of large urinary diversion calculi, this technique requires cystoscopy through the continent stoma to achieve percutaneous access under direct vision which has the potential for damage to the continence mechanism as well as the development of stomal stenosis and has only theoretical implications for access to the upper tract.

Smith et al. reported the first large series of percutaneous resections of UTTCC in a solitary kidney [40]. The oncologic efficacy of percutaneous resection has most often been measured in terms of disease recurrence, which has been shown to correlate with tumor grade [20, 41–43]. In review of several large series, recurrence rates for grade 1 (5–20%) [23, 41–44] and grade 2 diseases (6–33%) [23, 41–45] have been reported as significantly lower than recurrence rates for grade 3 disease (31–60%) [23, 41–43]. In addition, tumor grade has been shown to have prognostic significance, and death from low-grade UTTCC is rare [45]. It is important to note that prognosis for high-grade and high-stage UTTCCs is poor regardless of treatment modality. In a series of 25 patients undergoing percutaneous resection of grade 3 disease, Liatsikos et al. reported a 56% recurrence rate and 64% disease-specific survival [43] which is comparable to series examining ureteroscopic resection and radical therapy.

The major advantages of the percutaneous approach in patients following urinary diversion are that it allows direct access and the use of larger endoscopes, improving visualization. Both rigid and flexible endoscopes can be passed through the percutaneous tract, facilitating inspection of the entire calyceal system. The use of larger instruments facilitates the resection of large lesions, and makes tumor removal more efficient. The availability of larger instruments, including resectoscopes, grasping/biopsy forceps, and laser fibers, minimizes resection time allowing complete resection of large tumors in a single setting that would be difficult ureteroscopically [38]. Direct antegrade access also facilitates access to lower pole calyceal tumors. Ureteroscopic access and visualization of the lower pole are limited by the loss of deflection caused by instrument passage through the working channel [22]. The additional benefits of repeat nephroscopy for additional resection and the delivery of adjuvant therapy are facilitated by a percutaneous approach. This is of particular advantage in patients with large (>1 cm) tumor burden, solitary kidney, poor renal function, or significant comorbidites that would preclude open or laparoscopic nephroureterectomy. Bleeding due to the vascularity of the kidney and proximity to the hilum [46] and antegrade tract seeding [47] are complications of percutaneous treatment that despite infrequently being reported are still of significant concern. In comparison to the retrograde approach, percutaneous resection of upper tract TCC is more invasive and is associated with higher complication rates. Major complications of percutaneous resection include perforation, nephrostomy tract seeding, and bleeding. The incidence of blood loss varies among investigators and depends greatly on the size and extent of the treated lesion as well as ease of access, but transfusion rates up to 37% have been reported [43].

## 5. Adjuvant Therapy

A beneficial role for topical adjuvant therapy following the resection of UTTCC has not been proven in randomized trials. While retrograde instillation of agents into the collecting system has been described [35, 48], percutaneous resection with simultaneous nephrostomy tube placement facilitates antegrade instillation, maximizing chemotherapeutic agent contact with the urothelium. A disadvantage of retrograde catheterization, particularly in patients with continent urinary diversion, is that cystoscopy with ureteral catheter placement must be performed prior to each instillation.

Currently, there is no consensus as to which technique is more effective. In an initial study comparing outcomes in patients receiving postresection BCG with those who did not, Jabbour and Smith reported a significantly lower recurrence rate in patients with grade 1 tumors who received adjuvant BCG. There was no benefit for patients with grade 2 and grade 3 disease [38]. Rastinehad et al. [49] reported a 25% decreased likelihood of progression at 65 months follow up among 24 renal units with low-grade UTTCC undergoing BCG instillation. Despite the lack of evidence from randomized trials, the potential benefits and relative safety of adjuvant therapy provide an attractive alternative in patients with grade 2 and grade 3 disease in a solitary renal unit, patients with chronic renal insufficiency, or patients that are poor surgical candidates.

## 6. Conclusions

Nephroureterectomy is the gold standard treatment modality for high-grade and large-burden upper tract TCC recurrence following cystectomy and urinary diversion. However, with technical advances in equipment and increasing facility with endoscopic techniques, a minimally invasive approach is feasible in select patients. In the setting of a solitary kidney, chronic renal insufficiency, or significant comorbidity, preservation of renal function and prevention of recurrence are paramount. Reports of percutaneous tract seeding and recurrence due to pyelovenous backflow are uncommon but are a significant concern with each modality of conservative therapy. When choosing a surgical approach in a patient following lower urinary tract reconstruction, the ease of access, preservation of renal function, and oncologic efficacy must all be taken into consideration. Although technically possible, accessing the lower tract from a retrograde approach can be difficult and the capability for complete resection is limited for larger or lower pole lesions. Although more invasive, a percutaneous approach offers direct access with increased visualization, improved resection capability, and acceptable morbidity rates. Experience with ureteroscopic and percutaneous techniques enables full access to the reconstructed urinary tract and adds to the armamentarium of therapeutic options in the management of upper tract recurrence following cystectomy and urinary diversion.
